# Gastric perforation following improper cardiopulmonary resuscitation in out-of-hospital cardiac arrest

**DOI:** 10.12669/pjms.36.2.1363

**Published:** 2020

**Authors:** Guang-Ju Zhou, Ping Jin, Shou-Yin Jiang

**Affiliations:** 1 Guang-Ju Zhou, Department of Emergency Medicine, Second Affiliated Hospital, Zhejiang University School of Medicine; Research Institute of Emergency Medicine, Zhejiang University, Hangzhou 310009, China; 2 Ping Jin, Department of Emergency Medicine, Zhejiang Yuyao People’s Hospital, Yuyao 315400, China; 3 Shou-Yin Jiang. Department of Emergency Medicine, Second Affiliated Hospital, Zhejiang University School of Medicine; Research Institute of Emergency Medicine, Zhejiang University, Hangzhou 310009, China

**Keywords:** Gastric perforation, Cardiopulmonary resuscitation, Pneumoperitoneum, Case report

## Abstract

Gastric perforation is a rare complication of cardiopulmonary resuscitation (CPR), mostly resulting from incorrect airway management. If left unrecognized, it is associated with high mortality and morbidity. We present a case of gastric perforation after improper CPR. A 56-year-old drunken male was sent to the emergency department due to coma after fall onto the ground. He was thought to have cardiac arrest at scene and was saved with CPR maneuver by his friends who has never been trained before. He was taken to the hospital by emergency medical service personnel and presented with abdominal distention and extensive pneumoperitoneum. Emergency laparotomy was performed which revealed gastric perforation at the lesser curvature of the stomach. The laceration was repaired without any difficulty and the patient was discharged home without any neurological deficit. The aim of this report is to remind the public and emergency physicians that gastric perforation should be suspected in patients with distended abdomen and pneumoperitoneum after CPR. Because the most common risk factor for CPR-related gastric perforation is the bystander-provided resuscitation, it is encouraged for the public to take formal CPR training.

## INTRODUCTION

Cardiac arrest is a life-threatening medical condition affecting millions of individuals annually, which has been associated with poor survival and/or neurological outcome partly due to lack of public knowledge of cardiopulmonary resuscitation (CPR). While immediate high-quality CPR improves the chances of survival of cardiac arrest patients, improper CPR maneuvers can be detrimental.^1^ Moreover, it has been known that the quality of care provided during resuscitation attempts frequently does not meet the standards that the guidelines required due to lack of formal training.

Like any other medical interventions, CPR may be associated with some complications, and the overall incidence of CPR-related injuries in the literature ranges from 21% to 65%.^2^ These complications include rib and sternal fractures, pneumothorax and hydrothorax, cardiac contusion or laceration, hepatosplenic injuries, gastric mucosal lacerations, and rarely, gastric perforation.^2^ Although uncommon, if gastric perforation is left unrecognized, it will be associated with high mortality and morbidity. Here, we report a case of gastric perforation presenting with abdominal distention and pneumoperitoneum resulting from improper CPR.

## CASE REPORT

A 56-year-old male presenting with coma was brought to the emergency room in the morning. Forty minutes earlier, he suddenly fell onto the ground when drinking with his friends who found him to be unresponsive later. It was uncertain if he had spontaneous breathing and circulation at that time. His friends thought that he must have suffered sudden death and thus performed mouth-to-mouth rescue breathing with chest compression. In fact, his friends had never been trained on basic life support (BLS). Twenty minutes later, the emergency medical service personnel arrived, they assessed that the patient was unresponsive, with shallow breath and a pulse of 122 beats/min. He was immediately intubated and transported to the local hospital.

**Fig.1:**
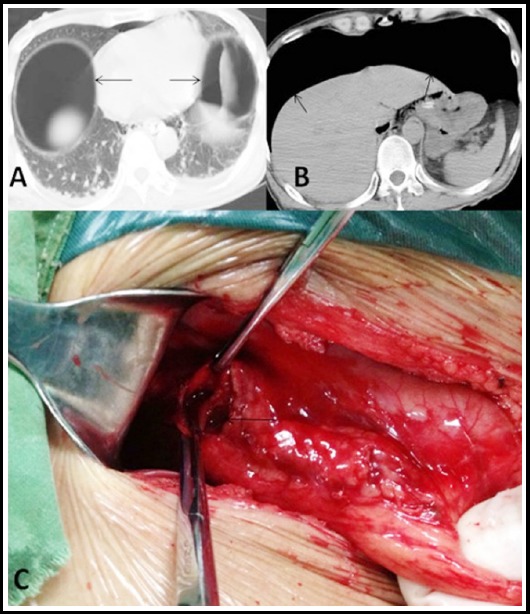
Computed tomography and intraoperative photograph of a patient with gastric perforation after improper cardiopulmonary resuscitation. A and B: Chest and abdomen CT scan show an appearance of gas (arrow) in the abdomen and the contractible stomach. C: A 2-cm laceration (arrow) is seen at the lesser curvature of the stomach.

Upon arrival, vital signs were blood pressure 92/61 mm Hg, pulse rate 125 beats/minutes, respiratory rate 26 breaths/min, and temperature 37.3 °C. Physical examination revealed abdominal distention, guarding, and absence of bowel sounds. Laboratory studies showed that the white blood cell count, hemoglobin, and blood glucose were all normal. FAST examination failed to assess the intra-abdominal organs due to gas accumulation in the abdomen. Cranial computed tomography (CT) scan was normal. Chest and abdomen CT scan revealed extensive pneumoperitoneum (Fig.1). Gastric perforation was suspected. A nasogastric tube was inserted and bloody fluid was drained. The patient received an urgent exploratory laparotomy. An upper midline incision was adopted, and a large amount of air was released when the abdomen cavity was opened. There was a 2-cm laceration at the lesser curvature of the stomach (Fig.1). The patient regained consciousness 10 hours after the operation and weaned from the mechanical ventilation. He was discharged home nine days later without any neurological deficits.

## DISCUSSION

Gastric rupture is a very rare complication after CPR. The incidence is estimated to be approximately one in every 1,000 cases, but as many as 12% of patients undergoing CPR have identifiable gastric lacerations.^3^,^4^ Pneumoperitoneum after CPR may be due to mediastinal air tracking into the peritoneal cavity via the diaphragmatichiatus or to gastric perforation. Abdominal distension not relieved by gastric decompression or a hemorrhagic gastric aspiration may be indicative of perforation, and emergency laparotomy should be considered. Studies have shown that the most common risk factor for CPR-related gastric perforation is bystander-provided BLS.^5^ Artificial ventilation without advanced airway and misplacement of endotracheal tube into the esophagus may cause large amount of air insufflating the gastrointestinal tract and lead to this severe complication. For the present case, the patient was not correctly evaluated for cardiac arrest by his friends and received wrong CPR with excessive ventilation, which resulted in gastric perforation.

CPR training efforts have undoubtedly saved thousands of lives. After educational intervention, the likelihood of survival with favorable neurological outcome of OHCA may be greatly improved. Thus, it is highly recommended that the society should establish a goal to train the public in standard CPR. The teaching of BLS has been recommended as a compulsory part of the school curriculum in many countries, and studies have shown that significant improvement was observed in students after BLS training.^6^ In 2004, the American Heart Association (AHA) recommended that American schools establish a goal to train all teachers and students in CPR. Now, many U.S. states and some European countries have included the teaching of BLS into the curriculum of high-school students, but in mainland China there is no current legislation that guarantees compulsory BLS training in schools.

For learners of non-medical personnel, it is highly recommended that strategies to teach CPR skills should be simplified, and AHA new recommendations make CPR easier to learn and to perform. Chest compression-CPR (CC-CPR), also called “Hands-only” CPR, has been proven to be at least as effective as conventional CPR for OHCA patients, it can provide critical perfusion to the heart and brain and is easier to perform for untrained lay-rescuers, it may also prevent defibrillable rhythm from degenerating into asystole.^7^-^9^ Because it is simple, easy to learn and master for lay rescuers, also because some rescuers are not willing to do mouth-to-mouth ventilation, the 2017 American Heart Association Focused Update on Adult Basic Life Support and Cardiopulmonary Resuscitation Quality recommend untrained lay rescuers should provide CC-CPR with or without dispatcher assistance.^10^ CC-CPR should be taught to all citizens as a minimum requirement for basic life support skills. For the present case, if the patient received CC-CPR, gastric perforation would not happen.

Since the knowledge of CPR and BLS programs are poorly educated among the public in mainland China, and the practice rate is also low in case of OHCA, steps should be taken to encourage public cardiopulmonary resuscitation in China.

## CONCLUSION

Gastric perforation should be suspected in patients with distended abdomen and pneumoperitoneum after CPR maneuvers. Because the most common risk factor for CPR-related gastric perforation is the bystander-provided resuscitation, it is encouraged for the public to take formal CPR training.
